# Fermentation metabolism and its evolution in algae

**DOI:** 10.3389/fpls.2013.00150

**Published:** 2013-05-22

**Authors:** Claudia Catalanotti, Wenqiang Yang, Matthew C. Posewitz, Arthur R. Grossman

**Affiliations:** ^1^Department of Plant Biology, Carnegie Institution for ScienceStanford, CA, USA; ^2^Department of Chemistry and Geochemistry, Colorado School of MinesGolden, CO, USA

**Keywords:** anoxic, anaerobiosis, hypoxic, fermentation, pyruvate metabolism

## Abstract

Fermentation or anoxic metabolism allows unicellular organisms to colonize environments that become anoxic. Free-living unicellular algae capable of a photoautotrophic lifestyle can also use a range of metabolic circuitry associated with different branches of fermentation metabolism. While algae that perform mixed-acid fermentation are widespread, the use of anaerobic respiration is more typical of eukaryotic heterotrophs. The occurrence of a core set of fermentation pathways among the algae provides insights into the evolutionary origins of these pathways, which were likely derived from a common ancestral eukaryote. Based on genomic, transcriptomic, and biochemical studies, anaerobic energy metabolism has been examined in more detail in *Chlamydomonas reinhardtii* (*Chlamydomonas*) than in any other photosynthetic protist. This green alga is metabolically flexible and can sustain energy generation and maintain cellular redox balance under a variety of different environmental conditions. Fermentation metabolism in *Chlamydomonas* appears to be highly controlled, and the flexible use of the different branches of fermentation metabolism has been demonstrated in studies of various metabolic mutants. Additionally, when *Chlamydomonas* ferments polysaccharides, it has the ability to eliminate part of the reductant (to sustain glycolysis) through the production of H_2_, a molecule that can be developed as a source of renewable energy. To date, little is known about the specific role(s) of the different branches of fermentation metabolism, how photosynthetic eukaryotes sense changes in environmental O_2_ levels, and the mechanisms involved in controlling these responses, at both the transcriptional and post-transcriptional levels. In this review, we focus on fermentation metabolism in *Chlamydomonas* and other protists, with only a brief discussion of plant fermentation when relevant, since it is thoroughly discussed in other articles in this volume.

## INTRODUCTION

### *Chlamydomonas* AS A MODEL ORGANISM

*Chlamydomonas reinhardtii* (*Chlamydomonas* throughout) is a soil-dwelling, unicellular green alga that is considered a model organism for studying photosynthetic energy metabolism, and the production of molecular hydrogen (H_2_) under anoxic conditions (Melis and Happe, 2001, 2004; Ghirardi et al., 2007). This alga has several metabolic features in common with those of vascular plants, although it also has structures and activities (e.g., flagella and eyespot) that were lost during vascular plant evolution. *Chlamydomonas* represents a robust system for probing biological processes with sophisticated molecular tools. The sequencing of all three *Chlamydomonas* genomes (nuclear, chloroplast, and mitochondrion; Lilly et al., 2002; Maul et al., 2002; Merchant et al., 2007) has facilitated the capture of information about gene and genome structure and potential regulatory sequences, including promoter regions, 3′- and 5′-UTRs and intron–exon junctions. Forward and reverse genetic screens have been developed to generate mutant strains with specific phenotypes, or that are disrupted for specific genes (Dent et al., 2005; Pootakham et al., 2010; Gonzalez-Ballester et al., 2011). Most information discussed in this manuscript on responses of algae to hypoxia/anoxia was derived from studies of *Chlamydomonas*, although information for other algae has been used to strengthen generalizations. Furthermore, we briefly discuss the evolution of the fermentation processes in prokaryotes and non-photosynthetic eukaryotes, but do not discuss plants since other contributions in this volume detail the responses of plants to hypoxic conditions.

### BASIC ENERGY-GENERATING PROCESSES

Whether in aerobic or anaerobic environments, the challenge for organisms to maintain viability can only be met if they can stay far from equilibrium. To achieve this situation, they must use energy to satisfy their metabolic demands, which includes continuous synthesis of the cellular energy currency (mostly ATP) along with maintenance of redox and ionic balances. Aerobic metabolism is used by several eukaryotic and prokaryotic organisms to efficiently synthesize ATP through oxidative phosphorylation; O_2_ serves as the terminal electron acceptor of the respiratory electron transport chain (Bailey-Serres and Chang, 2005). Nevertheless, life in low O_2_ (hypoxia) environments, or even in environments totally devoid of O_2_ (anoxia), is common on our planet. Diminished levels of O_2_ in various biotopes can result from geochemical or physical conditions, including flooding, excess rainfall, and winter ice encasement, but may also be a consequence of high metabolic activity of bacteria in habitats that are not well aerated. While anoxia is often transient, it can also be protracted, extending from diurnal periods, to months or years, and even to millennia or more (Grieshaber et al., 1994; Burnett, 1997; Danovaro et al., 2010). Furthermore, even though an organism may live in an oxic habitat, it may still perform anoxic metabolism under certain circumstances. For example, in the presence of sufficient levels of a fermentable substrate, many yeast strains will forego using O_2_ as a terminal electron acceptor and maintain vigorous fermentation of available substrates (van Dijken and Scheffers, 1986; Pronk et al., 1996).

### REDOX BALANCE THROUGH FERMENTATION

For cells to sustain viability during hypoxia/anoxia they must produce ATP and recycle the NAD(P)H and FADH_2_ generated by catabolic pathways, usually glycolysis. These compounds must be re-oxidized in a process involving the transfer of electrons to suitable terminal acceptor molecules, which are then typically secreted. Among eukaryotes, there are only two processes for maintaining redox balance and conserving energy when organisms experience anoxic conditions: (i) fermentation, which usually entails substrate-level phosphorylation (SLP), and (ii) anaerobic respiration which involves terminal electron acceptors like ${NO}_{3}^{-}$ and ${SO}_{4}^{2-}$ instead of O_2_ (Atteia et al., 2013). Anaerobic metabolism provides cells with low levels of chemical bond energy, generating ~2–3 ATP molecules per molecule of glucose metabolized; this compares to the over 30 ATP molecules generated by the oxidative metabolism of glucose.

## METABOLIC ENERGY GENERATION

### INTRODUCTORY REMARKS

Glycolysis oxidizes glucose to two molecules of pyruvate while generating two ATP molecules. During the oxidation of glucose there is also the production of two NADH molecules (four reducing equivalents). To maintain glycolytic flux and energy production, the cells must re-oxidize the NADH. In the absence of a functional TCA cycle under anaerobic conditions, *Chlamydomonas* places reducing equivalents into partially oxidized metabolic intermediates. The following section reviews the main anaerobic pathways activated in many organisms, including prokaryotic bacteria, eukaryotic fungi, and animals, when they are exposed to hypoxic/anoxic conditions.

### IN BACTERIA (Figure 1)

**FIGURE 1:**
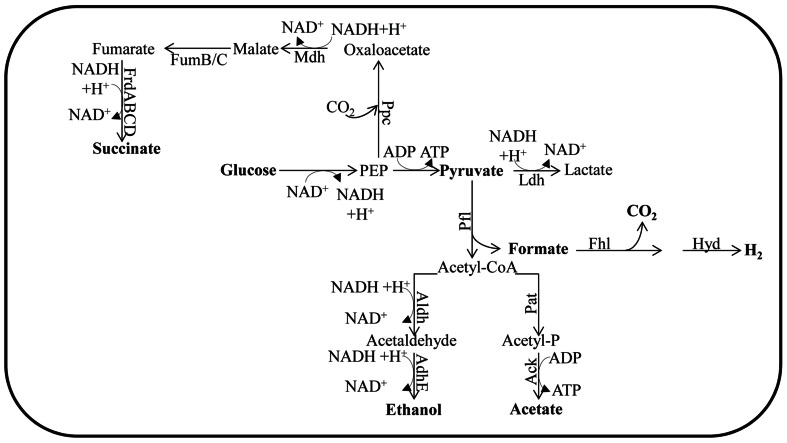
**Fermentation pathways of *E. coli***. The enzyme designations are: Ack for acetate kinase; AdhE for alcohol dehydrogenase; Aldh for aldehyde dehydrogenase; Fhl for formate hydrogen lyase; FrdABCD for fumarate reductase; FumB for fumarase B (anaerobic); FumC for fumarase C; Ldh for lactate dehydrogenase; Mdh for malate dehydrogenase; Pfl for pyruvate formate lyase; Ppc for phosphoenolpyruvate carboxylase and Pat for phosphotransacetylase. In *Chlamydomonas*, Aldh and AdhE activities are fused in the enzyme ADH1.

In the absence of O_2_ and under conditions that favor catabolite repression (e.g., excess glucose), *Escherichia coli* does not utilize a complete TCA cycle. However, it can use enzymes of this cycle to synthesize succinyl-CoA and 2-oxoglutarate; these metabolites represent the reductive and oxidative branches of the TCA cycle, respectively (Wolfe, 2005). This branched form of the TCA cycle does not generate energy but instead provides the precursor metabolites needed for cell viability. Therefore, ATP must come from glycolysis and SLP is associated with the phosphotransacetylase-acetate kinase pathway (Brown et al., 1977).

To sustain the flow of glycolytic metabolites when O_2_ availability severely limits aerobic respiration, the cells must re-oxidize NADH. In many bacteria the sugars are fermented to a mixture of ethanol and organic acids. This is achieved by reducing partially oxidized metabolic intermediates and forming, predominantly, the metabolites D-lactate, succinate, and ethanol, which are excreted into the environment along with formate and acetate (Wolfe, 2005; **Figure 1**). During anaerobiosis, pyruvate is the major metabolite synthesized as a consequence of glycolysis. The pyruvate can be converted to formate and acetyl-coenzyme A (acetyl-CoA) by pyruvate formate lyase (Pfl; Wolfe, 2005; **Figure 1**). This conversion is a non-oxidative reaction, which contrasts with oxidative decarboxylation that is mediated by the pyruvate dehydrogenase complex (Pdh, also sometimes designated Pdhc), which functions during respiratory metabolism. Pfl and its activating enzyme are widespread in facultative and obligate anaerobic eubacteria, as well as in archaea (Sawers and Watson, 1998). Mutants of *E. coli* devoid of Pfl do not grow anaerobically on glucose, but can grow if the medium is supplemented with acetate (Varenne et al., 1975). Under such conditions, *pfl* mutants maintain glycolytic ATP synthesis by reducing pyruvate to lactate. The generation of an *ldh* mutant in the *pfl* strain eliminates the remaining fermentation pathway for sustaining glycolysis. The formate derived from the Pfl reaction may be further metabolized to H_2_ and CO_2_ through the activity of formate hydrogen lyase (Fhl; Gottschalk, 1985) while the acetyl-CoA generated in this reaction can be converted to acetate or reduced to ethanol. Full conversion of acetyl-CoA to ethanol would not allow for redox balance since a single NADH is generated for each pyruvate that is synthesized from sugars, and two NADH molecules are required to convert pyruvate to ethanol. In order to achieve redox balance, *E. coli* must also synthesize additional products from the pyruvate, such as acetate and/or succinate (Dien et al., 2003).

The type and amount of fermentation end products excreted by bacteria, and the level of NADH generated for recycling, are highly dependent upon the substrate being metabolized by the bacterium. For example, bacteria using sorbitol, a highly reduced carbon compound, produce three NADH molecules per molecule of substrate, while a highly oxidized carbon compound such as glucuronic acid generates no NADH. To regenerate NAD^+^ from the NADH formed during the oxidation of sorbitol, bacteria synthesize and excrete ethanol (Wolfe, 2005). In contrast, cells growing on glucuronic acid are redox balanced and therefore no ethanol will be synthesized; instead, most pyruvate will be converted to acetate (Alam and Clark, 1989). The composition of excreted fermentation products also depends on the oxidation state of the cells and the pH of the medium. At neutral or higher pH, the main end products are acetate, ethanol, and formate, with moderate levels of succinate (Belaich and Belaich, 1976). However, as the pH becomes more acidic, cells produce lactate instead of acetate and formate (Bunch et al., 1997).

The conversion of acetyl-CoA to acetate is catalyzed by the phosphoacetyltransferase–acetate kinase (Pat–Ack also known as Pta-AckA) pathways. The Pat–Ack pathway generates one ATP per molecule of pyruvate metabolized, but consumes no NADH (**Figure 1**). In contrast, reduction of acetyl-CoA to ethanol is catalyzed by the bifunctional acetaldehyde/alcohol dehydrogenase (AdhE). While this reaction consumes reducing equivalents, it does not result in the generation of ATP (Wolfe, 2005). By coordinating the amount of ethanol and acetate (and other organic acids) synthesized and excreted into the medium, bacteria can efficiently balance their energy requirement with the need to recycle redox carriers (as reviewed by Wolfe, 2005).

There are two major acetate-producing pathways in *E. coli*; these are pyruvate oxidase (PoxB) and Pat–Ack (mentioned above). While PoxB decarboxylates pyruvate to acetate aerobically, the Pat–Ack complex is active under both aerobic and anaerobic conditions, converting acetyl-CoA to acetate (Hahm et al., 1994; Yang et al., 1999). The Pat–Ack reactions are sequential, reversible, and considered important for balancing the cellular carbon flux during exponential, aerobic and anaerobic growth (Chang et al., 1999; Avison et al., 2001). Pat converts acetyl-CoA and inorganic phosphate to acetyl phosphate (acetyl-P) and CoA, while Ack catalyzes the formation of ATP and acetate from acetyl-P and ADP (Rose et al., 1954). In *E. coli*, the *pat* and *ack* genes are organized in an operon (Kakuda et al., 1994). Mutants defective for Pat can neither synthesize acetate nor grow anaerobically (Gupta and Clark, 1989).

Under conditions in which anaerobically maintained *E. coli* cells are accumulating high levels of pyruvate or growing in a low pH medium, they can convert pyruvate to lactate through the activity of lactate dehydrogenase (Ldh; Clark, 1989; **Figure 1**). Alternatively, pyruvate or phosphoenolpyruvate (PEP) can be converted to a C4 intermediate of the TCA cycle by the catalytic addition of CO_2_ (Clark, 1989; **Figure 1**). In some cases, malic enzymes can carboxylate pyruvate forming malate, while phosphoenolpyruvate carboxylase (Ppc) can catalyze the formation of oxaloacetate (OAA) from PEP and CO_2_ (Clark, 1989; **Figure 1**). Both OAA and malate are then further reduced to succinate (Clark, 1989; **Figure 1**). This conversion is catalyzed by the sequential action of malate dehydrogenase (Mdh), fumarase (FumB and FumC), and fumarate reductase (FrdABCD; Clark, 1989; **Figure 1**); the gene encoding fumarase B is induced under anaerobic conditions (Woods et al., 1988). Since the amount of NADH generated varies with the nature of the substrate and the composition of the fermentation products generated, the redox balance and recycling of the NADH can be achieved by modulating the activities of the various fermentation pathways, which would result in a mix of end products, including ethanol, formate, acetate, and lactate (when necessary). Hence, *E. coli* mutants of *ldh* show no growth defects under anaerobic conditions because of compensatory pathways (Mat-Jan et al., 1989). Tarmy and Kaplan (1968) reported that fermentative Ldh is allosterically regulated and that its activity increases as the cellular pyruvate concentration increases; when pyruvate concentrations are low, the enzyme has very low activity. In contrast, *E. coli adhE* mutants do not synthesize alcohol dehydrogenase and cannot grow anaerobically on sorbitol, glucose, or gluconate since they cannot maintain redox balance, but they are able to ferment glucuronate (as reviewed by Clark, 1989).

### IN ALGAE (Figure 2)

**FIGURE 2:**
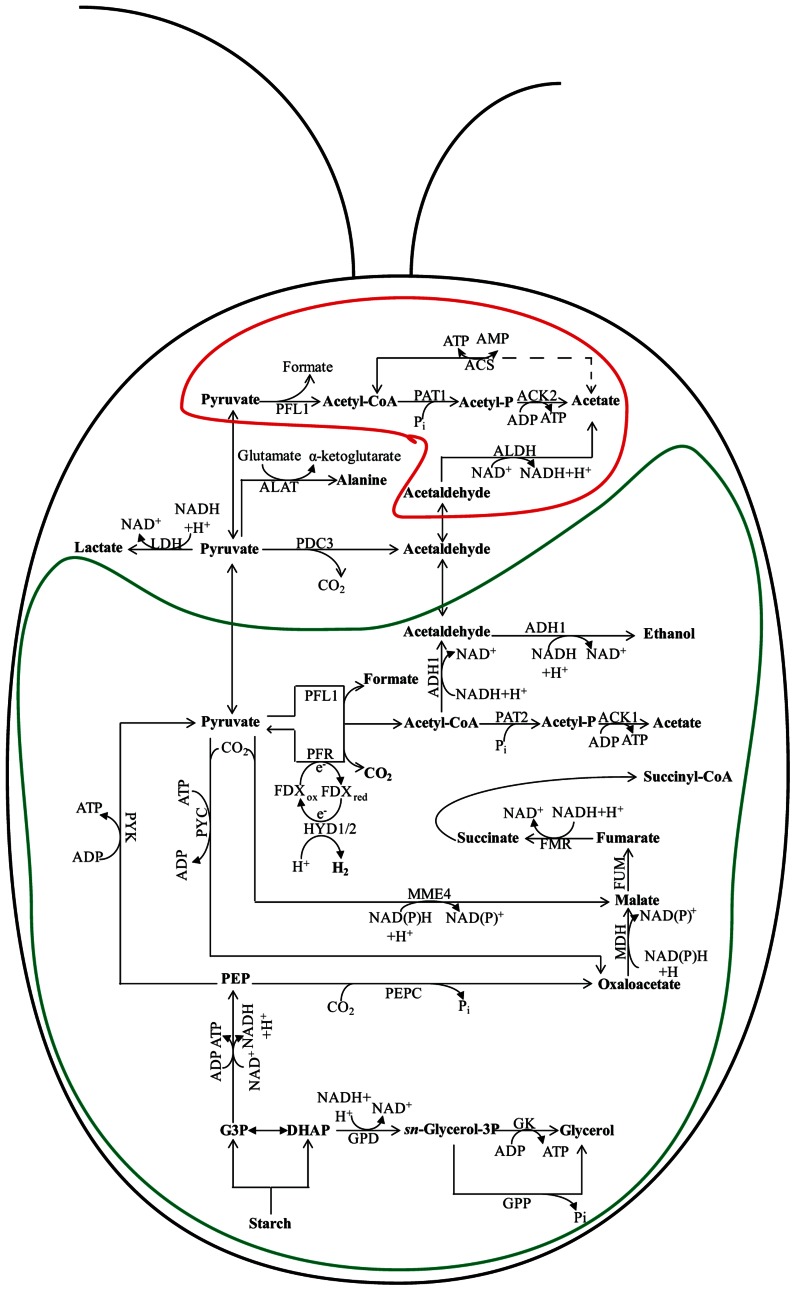
**Fermentation pathways in *Chlamydomonas***. In wild-type (WT) *Chlamydomonas* cells, the major fermentative products are formate, acetate, and ethanol, with CO_2_ and H_2_ generated as minor products. The pathway leading to fermentative succinate generation is not readily detected in WT cells grown under laboratory conditions, but becomes prominent in the *hydEF-1* mutant (Dubini et al., 2009). An increase in lactate production, which is almost undetectable in fermenting WT cells, is observed in the *pfl1* mutants (Philipps et al., 2011; Burgess et al., 2012; Catalanotti et al., 2012). Glycerol accumulation occurs in the *adh1* single and the *pfl1–1adh1* double mutants (Catalanotti et al., 2012; Magneschi et al., 2012). The protein designations in this figure are: FMR for fumarate reductase; MME4 for malic enzyme 4; PDC3 for pyruvate decarboxylase 3; PEPC for phosphoenolpyruvate carboxylase; PYC for pyruvate carboxylase; PYK for pyruvate kinase; PFR for pyruvate ferredoxin oxidoreductase; ACS for acetyl-CoA synthase; FDX for ferredoxin; HYDA1 and HYDA2 for the hydrogenases; GK for glycerol kinase; GPD for sn-glycerol-3 phosphate dehydrogenase and GPP for glycerol 3-phosphate phosphatase. See **Figure 1** for ACK1; ACK2; ADH1; ALDH; FUM; LDH; MDH; PAT1; and PAT2 designations. The enclosure delineated by a green line represents the chloroplast while the enclosure delineated by a red line represents the mitochondrion. The placement of some of the enzymes into specific subcellular compartments is speculative.

Fermentation of stored organic compounds by phototrophic microorganisms can represent a significant part of their overall energy budget as many of these ecologically important organisms spend much of their lifecycle under light-limited, hypoxic/anoxic conditions. Several species of water-oxidizing, photosynthetic algae can metabolize endogenous polysaccharides or secondary metabolites when the environment becomes anoxic, enabling them to generate the ATP necessary to drive metabolic and energy-requiring processes (Gfeller and Gibbs, 1984, 1985; Kreuzberg, 1984; Gibbs et al., 1986; Ohta et al., 1987). During dark fermentation, cellular polysaccharide reserves are catabolized, generating the needed ATP, while the co-produced NADH must be re-oxidized. The primary fermentation pathways used during anoxia vary among different algal species (Ohta et al., 1987; Atteia et al., 2013). Green algae such as *Chlamydomonas reinhardtii*, *Chlamydomonas moewusii*, *Chlorogonium elongatum*, and *Chlorella fusca* ferment starch to a variety of end products including acetate, ethanol, formate, glycerol, lactate, H_2_, and CO_2_ (Gaffron and Rubin, 1942; Ben-Amotz, 1975; Klein and Betz, 1978; Grossman et al., 2007; Mus et al., 2007). The heterofermentation patterns vary among green algal species (and sometimes among strains) and can also significantly vary with changes in environmental conditions, including the medium composition and carbon source. For *Chlamydomonas*, dark fermentation leads to the production of formate, acetate, and ethanol in a 2:1:1 ratio (Mus et al., 2007; **Figure 2**). In contrast, *Chlamydomonas moewusii* cells do not excrete formate during exposure to dark anoxic conditions; the major end products synthesized by this organism are acetate, glycerol, and ethanol (Klein and Betz, 1978; Meuser et al., 2009).

Some algae do not excrete fermentation products, but instead store them (reviewed by Müller et al., 2012; Atteia et al., 2013). *Euglena gracilis* synthesizes ATP when maintained under anoxic conditions with the concomitant accumulation of up to 60% fatty acids by dry weight (Inui et al., 1982). When the cells are returned to oxic conditions, the stored fatty acids can be converted back to acetyl-CoA, which can then be oxidized to CO_2_ or used to form paramylon reserves (Inui et al., 1982).

Diatoms and dinoflagellates are present in anoxic marine sediments (Jewson et al., 2006). The diatoms that inhabit these sediments accumulate high concentrations of nitrate (Lomstein et al., 1990), which is used as an electron acceptor in respiratory metabolism (e.g., generating ammonium) allowing these organisms to survive under dark anoxic condition (Kamp et al., 2011).

#### Enzymes of fermentation in chlamydomonas (Figure 2)

Currently, most information on fermentation metabolism in algae comes from studies of *Chlamydomonas* (Gfeller and Gibbs, 1984, 1985; Kreuzberg, 1984; Gibbs et al., 1986; Ohta et al., 1987; Hemschemeier and Happe, 2005; ; Mus et al., 2007; Hemschemeier et al., 2008; Dubini et al., 2009; Philipps et al., 2011; Burgess et al., 2012; Catalanotti et al., 2012; Magneschi et al., 2012). Genes encoding proteins associated with a diverse set of fermentative pathways have been identified on the *Chlamydomonas* genome, while a number of biochemical studies have revealed various fermentation circuits. The flexibility among the different pathways for catabolism of stored carbon under dark, anoxic conditions has been demonstrated through analyses of various mutants perturbed for these pathways (Mus et al., 2007; Dubini et al., 2009; Philipps et al., 2011; Burgess et al., 2012; Catalanotti et al., 2012; Magneschi et al., 2012). This flexibility allows *Chlamydomonas* to satisfy its energy requirements as O_2_ from the surrounding environment is depleted.

Over the course of the day there is a natural cycle for storage and utilization of fixed carbon. In phototrophic organisms, polysaccharides (sometimes lipids) accumulate in cells during daylight hours when photosynthetic CO_2_ fixation is a dominant metabolic process. During the evening, much of the starch reserve can be hydrolyzed to sugars by amylase activity (Ball, 1998; Dauvillee et al., 2001a,b; Zabawinski et al., 2001) and then, through the activity of glycolysis, be converted to pyruvate (**Figure 2**). As in bacteria, pyruvate fuels fermentation processes, serving as substrate for pathways that generate various organic acids, acetyl-CoA, alcohols, CO_2_, and H_2_. *Chlamydomonas* has multiple pathways for converting pyruvate to acetyl-CoA (Hemschemeier and Happe, 2005; Atteia et al., 2006; Grossman et al., 2007; see **Figure 2** for details). Three enzymes involved in these pathways are pyruvate formate lyase (PFL1), pyruvate ferredoxin oxidoreductase (PFR, often referred to as PFOR), and the pyruvate dehydrogenase (PDH) complex. As PDH generates NADH, a product that must be re-oxidized to sustain fermentation metabolism, it is presumed that PFL1 and PFR are the favored pathways for pyruvate catabolism in hypoxic/anoxic cells (**Figure 2**). While PFL1 catalyzes the conversion of pyruvate to acetyl-CoA and formate, in the PFR reaction pyruvate is oxidized to acetyl-CoA and CO_2_ with the concomitant generation of reduced ferredoxin (FDX). FDX can then pass reducing equivalents to hydrogenases to generate H_2_ (Happe and Naber, 1993; Ghirardi et al., 1997, 2000, 2007; Melis et al., 2000; Melis and Happe, 2001; Müller, 2003). However, the reduced FDX can also serve as a substrate for nitrite and sulfate/sulfite reductases (Ghirardi et al., 2008).

The acetyl-CoA produced by PFL1 and PFR reactions is either reduced to ethanol by alcohol/aldehyde dehydrogenase 1 (ADH1; Hemschemeier and Happe, 2005; Atteia et al., 2006; Dubini et al., 2009), or metabolized to acetate by the PAT–ACK (Atteia et al., 2006). An alternative pathway for ethanol production may be direct decarboxylation of pyruvate to CO_2_ and acetaldehyde through the activity of pyruvate decarboxylase (PDC3). The acetaldehyde generated in this reaction can be reduced to ethanol by ADH (either the same enzyme that catalyzes acetyl-CoA reduction or a distinct enzyme, e.g., ADH2). While the conversion of acetyl-CoA to ethanol by ADH1 oxidizes two NADH molecules, only a single NADH is oxidized in the PDC pathway.

Mutants in specific branches of fermentative metabolism have proven extremely valuable for elucidating the various routes of fermentation metabolism in *Chlamydomonas*, which are shown in **Figure 2**.

#### Formate production

Formate was demonstrated to be the dominant, secreted organic acid synthesized by *Chlamydomonas* maintained in anoxic conditions at near neutral pH in dark (Kreuzberg, 1984; Gibbs et al., 1986). The synthesis of formate by PFL1 uses a free-radical mechanism to catalyze the homolytic cleavage of pyruvate into formate and acetyl-CoA. This reaction depends upon a radical *S*-adenosyl methionine-dependent activating enzyme, designated PFL-AE (Atteia et al., 2006; Hemschemeier et al., 2008), which is usually present as an inactive form in aerobic cells, and is allosterically activated by pyruvate. In *Chlamydomonas*, PFL1 appears to be located in both mitochondria and chloroplasts (Kreuzberg et al., 1987; Atteia et al., 2006).

Algal strains deficient for PFL1 activity were isolated by independent groups (Philipps et al., 2011; Catalanotti et al., 2012) using different strategies (Burgess et al., 2012). The elimination of PFL1 activity in *Chlamydomonas* led to a marked accumulation of extracellular lactate, elevated pyruvate decarboxylation, and extracellular ethanol accumulation (**Figure 2**). The accumulation of lactate in the medium of *pfl1* mutants allows for recycling of NADH as a consequence of pyruvate reduction by LDH. Catalanotti et al. (2012) also demonstrated that the *pfl1* mutant accumulates elevated intracellular levels of lactate and alanine. Additionally increased intracellular levels of succinate, malate, and fumarate were observed, suggesting operation of the left branch of the reverse TCA reactions to recycle NADH.

#### Ethanol production

Acetyl-CoA produced by PFR/PFL1 activities can be metabolized to generate ATP by conversion to acetate or to help maintain redox balance by conversion to ethanol (Mus et al., 2007). *Chlamydomonas* possesses three distinct enzymes potentially important for ethanol production when the cells become anoxic: ADH1 (putative dual-function alcohol/acetaldehyde dehydrogenase; Mus et al., 2007; Hemschemeier et al., 2008; Magneschi et al., 2012), and two other putative alcohol dehydrogenases that were identified based on protein homology, designated ADH2 (Augustus version 5.0 protein identifier 516421) and ADH3 (Augustus version 5.0 protein identifier 516422). ADH1 has been localized to chloroplasts (Terashima et al., 2010).

A *Chlamydomonas* mutant devoid of ADH1 was unable to synthesis either ethanol or CO_2_ when the cells were transferred to anoxic conditions (Magneschi et al., 2012). The inability of the *adh1* mutant to accumulate ethanol and CO_2_, while synthesizing low levels of formate, suggests that the acetaldehyde synthesized by PDC3 and the acetyl-CoA synthesized by PFL1 and PFR cannot be rapidly reduced in the mutant. These findings also indicate that ADH1 is the only acetaldehyde-alcohol dehydrogenase in *Chlamydomonas* capable of reducing acetyl-CoA or acetaldehyde to ethanol under the conditions used in this study. Interestingly, the *adh1* strain was able to compensate for its inability to reduce acetyl-CoA or acetaldehyde to ethanol by reducing a significant amount of pyruvate to lactate. This elevated lactate accumulation was not as high as the level measured in *pfl1* (Philipps et al., 2011; Burgess et al., 2012; Catalanotti et al., 2012). However, the *adh1* mutants also accumulated high extracellular and intracellular levels of glycerol relative to anoxic wild-type (WT) cells. This acclimation response removes a significant amount of the C3 metabolites at the dihydroxyacetone phosphate (DHAP) step of the glycolytic pathway, which is prior to the reduction of NAD^+^ to NADH; the DHAP is then used as a substrate to re-oxidize NADH in the synthesis of glycerol (**Figure 2**).

#### Acetate production

The acetyl-CoA that is produced by PFL1 or PFR activities can be converted to acetate by PAT and ACK (Mus et al., 2007). Two parallel pathways have been identified in *Chlamydomonas*; PAT1–ACK2 appear to be mitochondrial while PAT2–ACK1 are in the chloroplast (Atteia et al., 2006, 2009; Terashima et al., 2011; **Figure 2**). Interestingly, the *PAT2* and *ACK1* genes are contiguous on the genome while *PAT1* and *ACK2* are far apart on the same chromosome (http://genome.jgi-psf.org/Chlre4/Chlre4.home.html).

While PAT–ACK activities comprise the predominant pathways for acetate formation under dark anaerobiosis, other enzymes are present on the *Chlamydomonas* genome that may play a role in acetate synthesis. Four genes encoding homologs of acetyl-CoA synthase (ACS) and eight genes encoding homologs of aldehyde dehydrogenase (ALDH) have been identified on the *Chlamydomonas* genome (Kirch et al., 2004, 2005; Brocker et al., 2012). The ACSs catalyze the putatively reversible conversion of acetate to acetyl-CoA (dash line in **Figure 2**). The ALDH reaction produces NAD(P)H during the conversion of acetaldehyde to acetate, therefore it is unlikely that these enzymes are active in fermentative metabolism when the cells require regeneration of reducing power (Kirch et al., 2004, 2005; Brocker et al., 2012). To date, there is no biochemical evidence to demonstrate that these alternative pathways for acetate generation are active in *Chlamydomonas*. In bacteria, the two pathways active under aerobic conditions that generate acetate are the Pat–Ack pathway, which is active in exponentially growing cells, and the PoxB pathway, which dominates during late exponential and stationary phase (Dittrich et al., 2005). It is uncertain whether or not similar regulatory features occur in *Chlamydomonas*.

The presence and/or production of acetate as *Chlamydomonas* cells become anoxic was found to be critical for maintenance of anoxic conditions in the light since acetate assimilation promotes O_2_ utilization (Kosourov et al., 2007; Morsy, 2011). The level of acetate accumulation during fermentative metabolism has proven to be difficult to predict, probably because it can also be used for the biosynthesis of key metabolites in anoxic cells, provided sufficient ATP and NAD(P)H is available. The *adh1* mutant exhibits a higher ratio of acetate production under anoxic conditions compared to WT cells, which reflects the elimination of ethanol production from the acetyl-CoA that is generated by PFL1 and PFR activities in the mutant strain; glycerol and lactate production serve as the primary NADH re-oxidation mechanisms in this mutant (Magneschi et al., 2012). In contrast, the *pfl1* mutant strains exhibit strongly reduced acetate accumulation (Burgess et al., 2012; Catalanotti et al., 2012); this decrease is likely due to a diminished intracellular acetyl-CoA pool.

#### H_2_ production

The mitochondria of cells maintained in aerobic conditions use PDH to convert pyruvate to acetyl-CoA; the acetyl-CoA generated can be metabolized to CO_2_ by the TCA cycle. In some animals PDH can function under anaerobic conditions (reviewed by Tielens and van Hellemond, 1998; Tielens et al., 2002; Hoffmeister et al., 2005; Tucci et al., 2010; Atteia et al., 2013). However, in many prokaryotes and eukaryotes, pyruvate oxidation in the absence of O_2_ is typically mediated by PFR. PFR belongs to a large family of thiamine pyrophosphate (TPP)-dependent enzymes. It catalyzes the oxidative cleavage of the carbon–carbon bond of the carboxyl group of pyruvate to liberate CO_2_ and reducing equivalents, with the attachment of the resulting acetyl group to CoA. However, unlike PDH, PFR can also function in the reverse direction catalyzing the production of pyruvate from CO_2_ and acetyl-CoA (Evans et al., 1966; see below), with FDX or flavodoxin serving as electron donors (Charon et al., 1999; Ragsdale, 2003; **Figure 2**). In *Chlamydomonas*, the reduced FDX generated from pyruvate oxidation by PFR activity can be re-oxidized by hydrogenases, generating H_2_ (Müller, 2003), or by reactions that enzymatically reduce nitrite and sulfate/sulfite. Hydrogenases are widespread among prokaryotes, whereas they are not as common among eukaryotes, and are restricted to a subset of unicellular eukaryotes, including photosynthetic algae (Meuser et al., 2011; Müller et al., 2012). *Chlamydomonas* hydrogenases belong to the class of [FeFe]-hydrogenases in which a [4Fe4S] cluster is linked through a cysteine residue to a 2Fe- cluster (Peters et al., 1998; Mulder et al., 2011).

**FIGURE 3:**
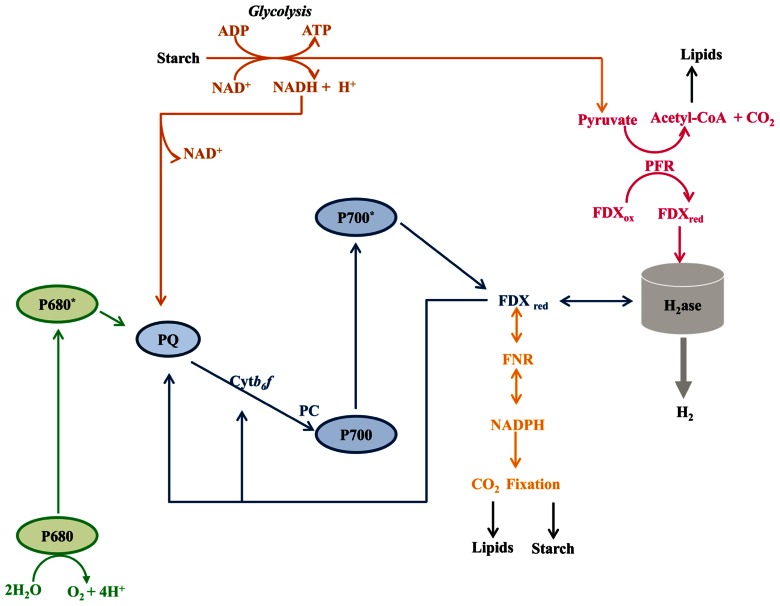
**Metabolic pathways associated with hydrogenase activity**. Five distinct metabolisms are depicted: (1) H_2_ production dependent on the complete photosynthetic electron transport system (PSII, PSI, FDX, H_2_ase; green and blue lines); (2) H_2_ production requiring starch catabolism and PSI activity (starch, glycolysis, PQ, PSI, FDX, H_2_ase; brown and blue lines); (3) H_2_ production in the dark from pyruvate oxidation (starch, glycolysis, pyruvate, FDX, H_2_ase; brown and magenta lines); (4) H_2_ oxidation coupled to CO_2_ reduction, with respiratory O_2_ uptake used to generate ATP (H_2_, H_2_ase, FDX, FNR, NAD(P)H, CO_2_ fixation; orange line); (5) H_2_ oxidation coupled to PSI-driven cyclic electron flow and ATP production (H_2_, H_2_ase, FDX, PSI, FDX and continued cycling; blue line). Abbreviations are: Cytb_6_f, cytochrome b_6_f complex; FDX, ferredoxin; FNR, ferredoxin NAD(P)^+^ reductase; H_2_ase, hydrogenase enzyme; PC, plastocyanin; PFR, pyruvate ferredoxin oxidoreductase; PQ, plastoquinone pool; P700, reaction center of photosystem I; P680, reaction center of photosystem II. For simplicity we have not tried to scale the energy potential of the electron carriers downstream of FDX.

Hydrogen production in algae is likely to have significant impacts on redox poising, photoprotection, and fermentative energy metabolism. Hydrogen production is coupled to cellular metabolism in a variety of ways, all of which are associated with O_2_ limitation: (i) direct biophotolysis, (ii) indirect biophotolysis, and (iii) dark fermentative metabolism (**Figure 3**). Direct biophotolysis involves light-dependent oxidation of water by photosystem II (PSII), the transfer of electrons from PSII to photosystem I (PSI), light-dependent excitation of PSI with the concomitant reduction of FDX and the subsequent transfer of electrons from FDX to hydrogenase (Benemann et al., 1973; Greenbaum, 1982; Happe and Naber, 1993; Miura, 1995; Ghirardi et al., 2007). During direct biophotolysis, the O_2_ generated by PSII must be reduced in order to prevent the accumulation of O_2_ to levels that would inhibit the hydrogenase. Indirect biophotolysis involves non-photochemical reduction of the PQ pool by NAD(P)H generated as a consequence of catabolic metabolism, followed by light-dependent FDX reduction by PSI and the subsequent transfer of electrons from FDX to hydrogenase (Cournac et al., 2000; Kosourov et al., 2003; Mus et al., 2005; Chochois et al., 2009). In the third H_2_-production pathway, starch catabolism provides electrons to the hydrogenases under dark fermentative conditions (Gfeller and Gibbs, 1984; Kreuzberg, 1984; Ohta et al., 1987; Happe et al., 1994; Ghirardi et al., 1997; Melis and Happe, 2001; Posewitz et al., 2004; Mus et al., 2007; Dubini et al., 2009).

Hydrogenases also function in H_2_ uptake, with two distinct uptake pathways described in *Chlamydomonas* (Gaffron, 1944; Kessler, 1974; Maione and Gibbs, 1986a,b; Chen and Gibbs, 1992a; **Figure 3**). In the first pathway, H_2_ oxidation and cyclic PSI activity in the light are linked to RuBisCO-mediated anaerobic CO_2_ fixation. Electrons from H_2_ are used to reduce FDX, which then reduces FDX-NAD(P) oxidoreductase (FNR), leading to the generation of NAD(P)H which, along with the ATP generated by cyclic electron flow, can be used to fix CO_2_. This pathway requires the absence of O_2_ evolution from PSII. In the second pathway, termed the oxyhydrogen reaction, H_2_ oxidation occurs concomitantly with the uptake of low levels of O_2_ in a process that can be coupled to CO_2_ fixation (Gaffron and Rubin, 1942; Gaffron, 1944; Russell and Gibbs, 1968; Kessler, 1974; Chen and Gibbs, 1992a). Although not well characterized, it is posited that H_2_ oxidation provides the reducing equivalents for CO_2_ fixation and that the low levels of O_2_ present are respired to provide ATP (Gaffron and Rubin, 1942; Gaffron, 1944; Maione and Gibbs, 1986a; Chen and Gibbs, 1992a).

Recently, mutants were obtained in each of the two *HYDA* genes of *Chlamydomonas*, *HYDA1* and *HYDA2* (Meuser et al., 2012). The phenotypes of the single (*hydA1* and *hydA2*) and double (*hydA1–hydA2*) mutants were analyzed under both light and dark anoxic conditions. Both single mutants could catalyze H_2_ production from reductant generated from either fermentative or photosynthetic metabolism. However, the contribution of the HYDA2 enzyme to H_2_ photoproduction under the conditions tested was approximately 25% of that of HYDA1 (Godman et al., 2010; Meuser et al., 2012).

The impact of the *hydEF-1* lesion on fermentation is more interesting since it demonstrates the flexibility of *Chlamydomonas* anaerobic metabolism (see below). This mutant is unable to assemble the inorganic constituents of the hydrogenase active site, and consequently cannot catalyze H_2_ synthesis (Posewitz et al., 2004).

#### Succinate production

Anoxic cultures of the *Chlamydomonas* *hydEF-1* mutant exhibit lower CO_2_ evolution and reduced extracellular formate, acetate, and ethanol accumulation. Interestingly, the mutant synthesizes elevated levels of extracellular succinate (Dubini et al., 2009; **Figure 2**), indicating activation of a fermentative pathway that is not operating at significant levels in WT cells. Microarray data and metabolite analyses suggest that carboxylation of pyruvate in the *hydEF-1* mutant leads to the synthesis of either malate or OAA (or both), which is subsequently converted to succinate via reverse reactions of the TCA cycle. Activation of the reductive TCA branch as a means of recycling NADH was previously observed in anaerobic bacteria (Gray and Gest, 1965; Schauder et al., 1987; Buchanan and Arnon, 1990; Beh et al., 1993; Yoon et al., 1999), in the green alga *Selenastrum minutum* (Vanlerberghe et al., 1989, 1990) and in vascular plants (Sweetlove et al., 2010), but was not known to occur in *Chlamydomonas*.

The alternative pathway suggested by Dubini et al. (2009) not only explains succinate accumulation under anaerobic conditions, but also raises the possibility that *Chlamydomonas* could potentially operate a complete reverse TCA cycle. This would require that PFR functions in the direction of pyruvate synthesis under the appropriate metabolic conditions. Various researchers have suggested that PFR could function in the synthesis of pyruvate in *Chlamydomonas* (Chen and Gibbs, 1992b; Melis et al., 2007; Terashima et al., 2011; **Figure 2**). Chen and Gibbs (1992b) detected ATP-citrate lyase, as well as PFR and α-ketoglutarate synthase activities in *Chlamydomonas* cell extracts, speculating that the existence of these three key enzyme activities indicated that the reverse TCA cycle could operate in *Chlamydomonas*. These authors showed that a *Chlamydomonas* mutant with a compromised Calvin–Benson cycle takes up CO_2_ in the dark under minimal aerobic conditions (1% O_2_), and that the CO_2_ uptake is coupled to H_2_ oxidation (Chen and Gibbs, 1992a), suggesting that the reverse TCA cycle could be a significant pathway for CO_2_ assimilation when the Calvin–Benson cycle is compromised (Chen and Gibbs, 1992b). Hence, under these conditions H_2_ oxidation would provide the reducing equivalents to drive the reverse TCA cycle and to allow PFR to synthesize pyruvate, leading to the accumulation of an array of biosynthetic precursors. The possibility of PFR-dependent synthesis of pyruvate has also been observed in many hydrogenosome-containing eukaryotic organisms experiencing anaerobic conditions (Lindmark and Müller, 1973). Furthermore, in the unicellular microaerophilic eukaryote *Trichomonas vaginalis*, MME and PFR are central to carbohydrate metabolism in the hydrogenosomes (Müller, 1993). In addition, PFR and MME activities have been linked to malate production in the hyperthermophilic archaeon *Thermococcus kodakaraensis* KOD1, also suggesting reductive carboxylic acid cycle activity (Fukuda et al., 2005). The association of PFR and MME with pyruvate metabolism in hydrogenosome-containing anaerobic eukaryotes, the findings that a similar set of anoxic-induced proteins are associated with *Chlamydomonas* chloroplasts, and the metabolite data obtained with various *Chlamydomonas* strains exposed to anoxic, reductant-rich conditions, all suggest that the TCA cycle may operate in the reverse direction in *Chlamydomonas* chloroplasts in anoxic cells that have sufficient reducing equivalents and ATP.

#### Lactate and glycerol production

Glycerol and lactate are usually minor end products of green algal fermentation (Gfeller and Gibbs, 1984; Kreuzberg, 1984). Glycerol is synthesized from DHAP, and its synthesis results in recycling of one NADH. The reaction precedes the formation of pyruvate and the C3 oxidation (NADH formation) step in glycolysis. Hence, glycerol and lactate production in the *adh1* mutant would allow for efficient recycling of NADH, maintenance of redox balance and sustained glycolytic production of ATP even though the cells are unable to reduce acetaldehyde or acetyl-CoA to ethanol (Magneschi et al., 2012; **Figure 2**). The *pfl1–1adh1* double mutant cannot synthesize either formate or ethanol (Catalanotti et al., 2012; **Figure 2**). This strain, like *pfl1*, secretes significant levels of lactate, however, like the *adh1* mutant, it also synthesizes and secretes high levels of glycerol and acetate. Hence, this mutant exhibits a complete rerouting of glycolytic carbon to lactate and glycerol, transforming *Chlamydomonas* cells from a formate/acetate/ethanol to a glycerol/lactate fermenter (Catalanotti et al., 2012; **Figure 2**).

### IN OTHER EUKARYOTES

Eukaryotes specialized to thrive under aerobic conditions generally possess simple cytosolic fermentation pathways that enable them to tolerate short-term anoxia; these pathways facilitate accumulation of end products such as lactate, ethanol, and glycerol (reviewed by Müller et al., 2012). Some eukaryotes, including many algae, experience frequent exposure to anoxic conditions, where they are unable to use O_2_ as a terminal electron acceptor. These organisms have evolved a modest set of energy-generating pathways, which are reviewed below.

#### Ethanol, lactate, and glycerol fermentation (Figures 4 and 5)

**FIGURE 4:**
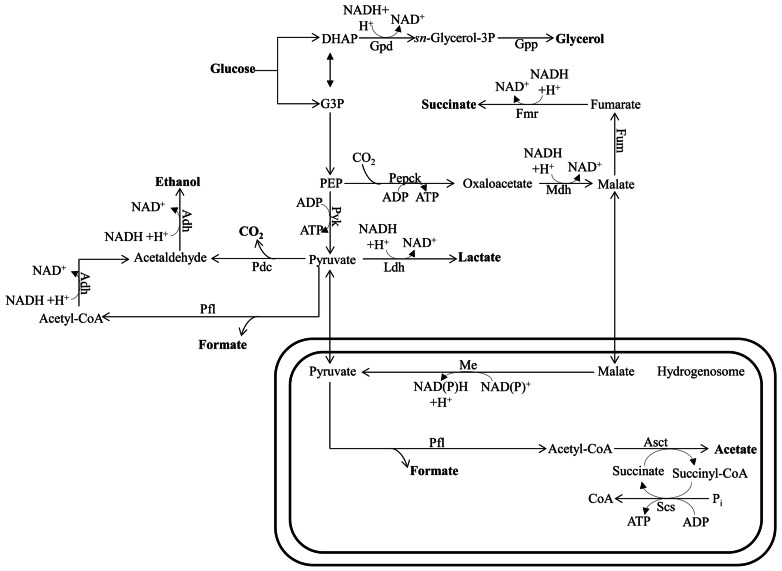
**Mixed-acid fermentative metabolism of the hydrogenosome-bearing anaerobic chytridiomycete fungus *Piromyces***. The circuitry is drawn based on data reported by Wang et al. (2001) and Boxma et al. (2004). This fungus uses pyruvate formate lyase for pyruvate catabolism in their hydrogenosomes. Glucose can also be metabolized in the cytosol to the end products succinate, lactate, formate, and ethanol. Bifunctional alcohol dehydrogenase (Adh), having both alcohol dehydrogenase and acetaldehyde dehydrogenase activities, mediates the cytosolic formation of ethanol. The protein designations in this figure are: Asct for acetate succinyl-CoA-transferase; Me for malic enzyme; Pepck for phosphoenolpyruvate carboxykinase; Scs for succinyl-CoA synthase; See **Figure 1** for Adh; Fum; Ldh; Mdh; and Pfl designations and **Figure 2** for Fmr; Gpd; Gpp; and Pdc designations. DHAP, dihydroxyacetone phosphate; G3P, glycerol-3-phosphate; PEP, phosphoenolpyruvate.

**FIGURE 5:**
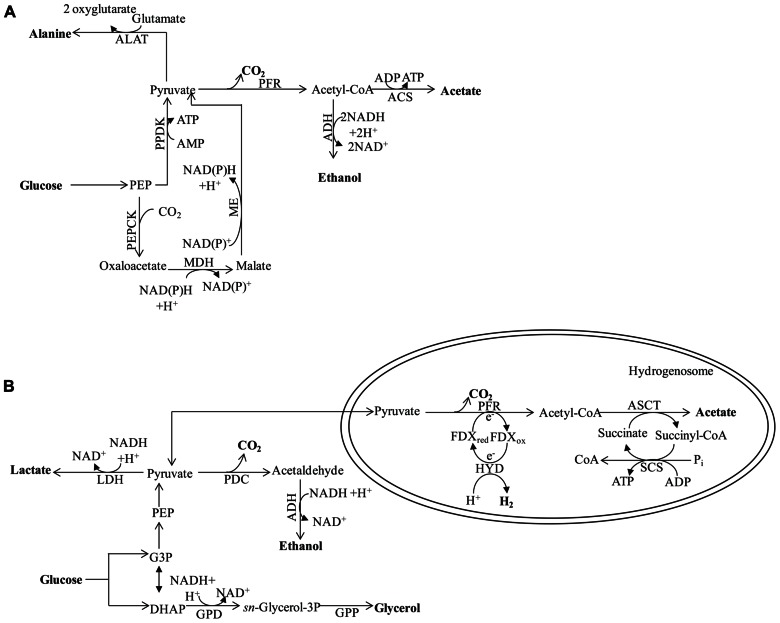
**(A)** Major metabolic pathways in the anaerobic intestinal parasite *Entamoeba histolytica*. The map is adapted from Müller et al. (2012). The energy metabolic pathways are localized in the cytosol. Pyruvate ferredoxin oxidoreductase is used for pyruvate decarboxylation/oxidation, ATP is synthesized through SLP via acetyl-CoA synthase (ADP forming). **(B)** Major pathways of anaerobic, molecular H_2_-producing, fermentative metabolism in *Trichomonas vaginalis*. Hydrogenosomal pyruvate breakdown involves PFR and functional [FeFe]-hydrogenase (HYD) in *Trichomonas*. Additional major end products of cytosolic fermentation in *Tvaginalis vaginalis* include alanine, lactate, ethanol, and glycerol. The protein designations in this figure are: ALAT for alanine aminotransferase and PPDK for pyruvate:orthophosphate dikinase. See **Figures 1–3** for the other protein designations.

When O_2_ in the environment is depleted, plants can use PDC to convert pyruvate to acetaldehyde, which is metabolized to ethanol by ADH (Gibbs and Greenway, 2003; Bailey-Serres and Voesenek, 2008). The ethanol generated in plant roots can rapidly diffuse into the rhizosphere, which limits its toxicity. Plants can also synthesize lactate under conditions of low O_2_. The transition from lactic to ethanolic fermentation appears to be controlled by the pH of the cytoplasm of the cell. A ~0.6 unit decrease in cytosolic pH favors PDC activity, which promotes ethanol production and limits lactate synthesis (reviewed by Bailey-Serres and Voesenek, 2008). This lactic to ethanolic switch is critical for maintaining cytosolic pH (Roberts et al., 1989). In addition to eliciting metabolic changes, low O_2_ can trigger alterations in plant morphology which include petiole or internode elongation, altered anatomy and cell ultrastructure in leaves and roots, development of lateral or adventitious roots and the formation of aerenchyma cells (Bailey-Serres et al., 2012).

Ethanol, lactate, and glycerol are common end products of fermentative metabolism in many organisms. The synthesis and excretion of ethanol allows carp to survive anaerobiosis for up to ~5 months (van Warde et al., 1993) and goldfish to withstand anoxia for several weeks (van den Thillart et al., 1983). It is notable that the fermentation pathways used for these reactions appear to have their origins in a typical yeast-type PDC and ADH (van Warde et al., 1993; **Figure 4**). The fungi, a highly diverse group, can also ferment carbohydrates to lactate, glycerol, and ethanol. Glycerol acts as a redox valve under anaerobic conditions since it enables re-oxidation of NADH that is generated during the conversion of sugars into biomass. While fungi may also excrete organic acids, the levels are generally low; these acids include formate, acetate, lactate, and succinate (**Figure 4**). Formate production is not uncommon in fungi as a result of the activity of a cytosolic (and hydrogenosomal) PFL, which provides the acetyl-CoA for ethanol production (Boxma et al., 2004; **Figure 4**).

Finally, pathogenic amoebozoa such as *Entamoeba histolytica* often experience anaerobic conditions; their main end products of anaerobic energy metabolism are alanine, CO_2_, ethanol, and acetate (Müller et al., 2012). The enzymes responsible for generating these products are exclusively in the cytosol (Müller, 2003). The initial reactions of the pathway involve conversion of PEP to pyruvate by pyruvate orthophosphate dikinase (PPDK; Reeves, 1968; **Figure 5A**), which also generates ATP. The pyruvate is then oxidized via PFR to CO_2_ and acetyl-CoA, with the latter converted into a mixture of acetate and ethanol (**Figure 5A**). Alternatively the PEP can be carboxylated to OAA by PEP carboxytransferase, reduced to malate by malate dehydrogenase (MDH) and the malate then converted to pyruvate by the malic enzyme (ME; **Figure 5A**; as reviewed by Müller et al., 2012). *Entamoeba* possesses a bifunctional aldehyde/alcohol dehydrogenase (ADH), which represents a fusion protein that contains an N-terminal aldehyde dehydrogenase domain and a C-terminal alcohol dehydrogenase domain. This enzyme regenerates 2 NAD^+^, is present in many eukaryotes, and has been found to be highly expressed in *Chlamydomonas* (Mus et al., 2007; Catalanotti et al., 2012; Magneschi et al., 2012), other green algae and protozoan parasites such as *Giardia intestinalis*, *Trichomonas*, and euglenids.

*Trichomonas vaginalis* synthesizes ethanol from pyruvate in the cytosol via PDC and ADH. However, the main end products of *T. vaginalis* fermentative metabolism are distributed between the cytosol (glycerol, lactate, and ethanol) and the hydrogenosome (CO_2_, H_2_, and acetate; **Figure 5B**). Similarly, *Chlamydomonas* can synthesize glycerol from DHAP, which is catalyzed by glycerol-3-phosphate dehydrogenase and glycerol-3-phosphatase, and LDH can catalyze lactate accumulation. However, while both reactions in *T. vaginalis* occur in the cytosol, their locations in *Chlamydomonas* are not clear.

#### Malate dismutation and acetate and propionate production (Figure 6)

**FIGURE 6:**
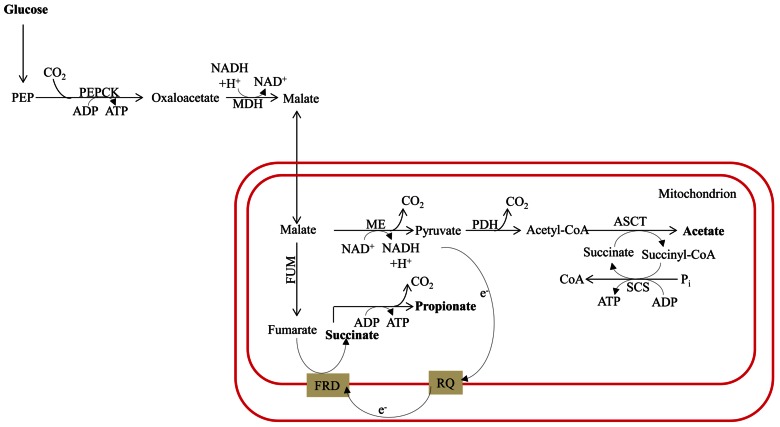
**Malate dismutation and energy metabolism in anaerobic mitochondria**. The map is redrawn based on the review of Müller et al. (2012). The main end products are acetate and propionate, with minor amounts of succinate. The designations in this figure are: FRD for fumarate reductase; PDH for pyruvate dehydrogenase complex; and RQ for rhodoquinone. See previous figure for other designations.

Fermentation in animals often involves malate dismutation. It is not uncommon for parasitic worms to switch to complete anaerobic metabolism once they are established in the host tissue. In parasitic mode they convert the PEP generated by glycolysis to OAA, which is then reduced to malate via a cytosolic malate dehydrogenase (**Figure 6**). This reaction results in re-oxidation of one molecule of NADH. The malate is then imported into the mitochondrion where dismutation occurs; a portion of the malate is oxidized to acetate (via pyruvate), and another is reduced to succinate. In the latter reaction, malate is converted to fumarate by FUM and the fumarate is reduced to succinate (**Figure 6**); this pathway is similar to the alternative fermentation pathway activated in the *Chlamydomonas* *hydEF-1* mutant. Many organisms excrete succinate produced by malate dismutation rather than decarboxylating the succinate to generate propionate plus an extra molecule of ATP (Pietrzak and Saz, 1981; Müller et al., 2012; **Figure 6**). Interestingly, in the parasite system, fumarate reduction is performed by a membrane-associated, anaerobiosis-specific enzyme (FRD) that is coupled to an electron transport chain that functions specifically under anaerobic conditions. Electrons are transferred from NADH to fumarate via rhodoquinone (RQ; **Figure 6**) instead of ubiquinone (UQ, which is normally used under oxic conditions); the lower redox potential of RQ (relative to UQ) allows for the thermodynamically favorable use of electrons in the synthesis of succinate by FRD (as reviewed by Müller et al., 2012).

#### Intracellular metabolite accumulation

O_2_ deficiency is often associated with a wide range of excreted metabolites, but may also trigger more complicated responses involving sequestration of specific end products. Plants experiencing low O_2_ accumulate alanine and γ-aminobutyric acid (GABA; reviewed by Bailey-Serres and Voesenek, 2008). Upon re-oxygenation, alanine can be recycled back to pyruvate, and GABA can be converted to succinate. This set of amino acid oxidation reactions may minimize the decline in cytosolic pH and reduce the loss of fixed carbon as ethanol or lactate. Alanine also accumulates in *T. vaginalis* and in many animals belonging to the Excavata taxa (Edwards et al., 1989) as a minor end product.

A less common fermentation process, but largely used in marine environments, involves opine formation. This pathway is localized to the cytosol and involves pyruvate condensation with an amino acid in a redox reaction that regenerates NAD^+^. A possible advantage of this alternative pathway for balancing cellular redox is that opine is less acidic than lactate. Moreover the process maintains an osmotic equilibrium since one amino acid is consumed per opine synthesized (Ballantyne, 2004).

#### Denitrification (Figure 7)

**FIGURE 7:**
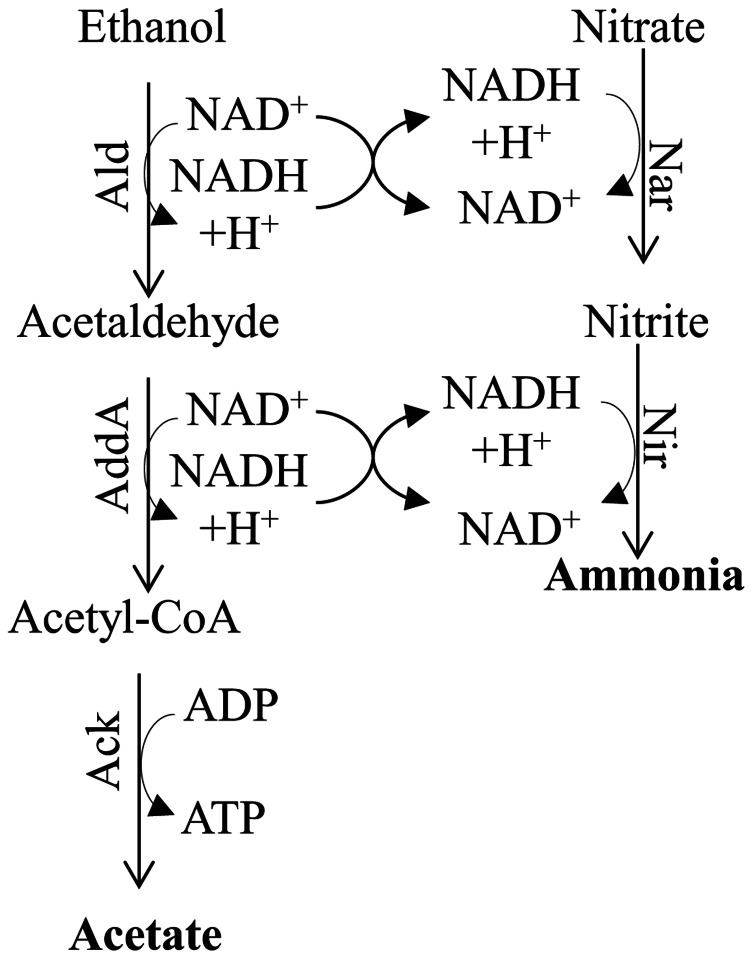
**Metabolic pathway for ammonia fermentation coupled to acetogenic oxidation of ethanol and substrate-level phosphorylation**. The protein designations in this figure are: Ald, alcohol dehydrogenase; Adda, acetaldehyde dehydrogenase; Nar, nitrate reductase; and Nir, nitrite reductase.

The capacity for nitrate respiration is widespread among bacteria, fungi, and other eukaryotic organisms (Morozkina and Kurakov, 2007). Details of the denitrification pathway have been studied in fungi and bacteria (see previous paragraphs). On the other hand, the enzymes required for nitrogen metabolism in foraminifera and diatoms are not well characterized, although the occurrence of the pathway was noted (Risgaard-Petersen et al., 2006; Kamp et al., 2011).

Numerous reports have demonstrated the presence of two main pathways for denitrification; one is localized in the mitochondrion and usually occurs under low O_2_ conditions, while the other, often referred to as ammonia fermentation, is localized in the cytosol (Zhou et al., 2001; Takasaki et al., 2004; Morozkina and Kurakov, 2007; **Figure 7**). The latter pathway appears to be activated under strict anoxic conditions and involves reduction of nitrate to ammonia using reductant generated by the catabolic oxidation of ethanol (the donor of electrons) to acetate, which is coupled to SLP. As shown in **Figure 7**, the ethanol is oxidized to acetaldehyde by an alcohol dehydrogenase (designated Ald), which is converted to acetyl-CoA by acetaldehyde dehydrogenase (AddA). The acetyl-CoA is then converted to acetate and CoA, with the concomitant production of ATP by Ack (Zhou et al., 2001). Under hypoxic conditions the ethanol is oxidized to acetate and the electrons generated in the reaction are used to reduce nitrite to N_2_O, which is excreted from cells (Zhou et al., 2001; **Figure 7**). Nitrate and nitrite reductases catalyze the reduction of nitrogen oxides to ammonia using NADH as the electron donor, and are assimilatory enzymes.

#### H_2_ and CO_2_ production

H_2_ and CO_2_ are generated in an ancestral anaerobic pathway that is present in many green algae. The generation of H_2_ in algae often serves as a redox valve. This pathway can be in chloroplasts, as in *Chlamydomonas* and other algae, in mitochondria-like organelles, as in the Stramenopiles, or in the hydrogenosomes of the amoebozoa, some opisthokonta and Excavata. H_2_ production is often associated with PFR activity, which oxidizes pyruvate to acetyl-CoA and CO_2_. Reduced ferredoxin transfers electrons to a hydrogenase that can convert protons and electrons into H_2_ (**Figure 5B**).

## METABOLITE PARTITIONING, ORGANELLE COMMUNICATION AND ITS EVOLUTION

### CARBON PARTITIONING BETWEEN ORGANELLES

Glycolysis is the backbone of eukaryote carbon and energy metabolism, leading to the production of pyruvate, ATP, and NADH. Further metabolism of the pyruvate can occur in the cytosol, mitochondrion, or plastid. For some eukaryotic organisms fermentation occurs entirely in the cytosol; the organisms included in this group are the protistan parasites such as *Giardia* and *Entamoeba* (Müller, 1996). Fermentations can also occur partly in hydrogenosomes, as is the case for *Trichomonas* (Müller, 1993). Among animals, fermentation often entails malate dismutation, involving segments of the mitochondrial electron transport chain, as in the case of the anaerobic mitochondria of many marine invertebrates and parasitic worms (Tielens et al., 2002; Tielens and van Hellemond, 2009).

A number of metabolic reactions can occur in more than one compartment in the cell and some enzymes may be routed to more than one cellular location; one example of this is PFL, which appears to occur in both chloroplasts and mitochondria, but dual localizations of proteins is not uncommon in eukaryotes (Atteia et al., 2006; Martin, 2010; Müller et al., 2012). Examining the network of activities in *Chlamydomonas* exposed to anoxic conditions raises some fundamental questions; one very important question is “How can an entire metabolic pathway be transferred to a new compartment?” This issue is still far from being resolved and more detailed biochemical and evolutionary analyses are necessary. However, it is becoming evident that over evolutionary time, enzymes and pathway can readily undergo re-compartmentation among subcellular locations in the cell including the mitochondrion, cytosol, hydrogenosome, and chloroplast. Small changes in targeting sequences might result in mistargeting, which could explain how individual activities, as well as entire pathways are found in more than one cellular compartment (Martin, 2010).

### EVOLUTIONARY INSIGHTS

This review presents information indicating that overall, the different groups of eukaryotic organisms share the same core pathways for hypoxic/anoxic energy metabolism. Although distinct mechanisms are used by obligate and facultative anaerobes, there is a certain set of enzymes consistently associated with fermentation metabolism among a variety of organisms ranging from the bacteria to algae, fungi, and metazoans. The heterofermentation that is associated with the algae differs from lactate or ethanol homofermentation that occurs in yeast and various multicellular organisms including plants and animals; fermentation patterns in *Chlamydomonas* show some similarities to mixed-acid fermentation, which is common in the enteric bacteria (Neidhardt et al., 1990). The *Chlamydomonas* genome appears to contain a complete (or near complete) spectrum of genes involved in anaerobic energy metabolism across all eukaryotes (Müller et al., 2012; Atteia et al., 2013). However, the ancestry of these genes, whether from single or multiple origins, remains to be established. Müller et al. (2012) in a recent review favor the hypothesis that many of the enzymes associated with anaerobic energy metabolism in eukaryotes share a common ancestor, which is supported by the finding that different eukaryotic lineages possess different subsets of the same ancestral collection of genes. Furthermore, if the various genes for anaerobic metabolism in protists were derived from multiple ancestral genes, then evidence for the lateral transfer of genes from multiple sources should be apparent. The fact that no eukaryotes perform sulfate reduction, ammonium oxidation, or methane oxidation suggests that the independent lateral transfer of anoxic pathway genes to eukaryotes is not a common occurrence. Instead, different lineages of eukaryotic anaerobes use distinct enzyme combinations selected from a limited core inventory of fermentative pathways. Although a common origin of these pathways is speculative at this point, Müller et al. (2012) observes that there is no pattern of lineage specific acquisition, and it remains unclear why alternative anoxic strategies are not widely observed in eukaryotes.

## CONCLUSION

*Chlamydomonas* is a metabolically versatile organism that can perform photosynthetic CO_2_ fixation, aerobic respiration, and anaerobic fermentation. This alga has served as a model system to examine many aspects of photosynthetic metabolism and recently has been used in studies of anaerobic metabolism; these latter studies have shown that *Chlamydomonas* contains a large and complex repertoire of anaerobic enzymes that are distributed among the different compartments of the cell. Initial characterizations have demonstrated that *Chlamydomonas* has flexible, mixed-acid fermentation, with features common to bacterial-, plant-, and yeast-type fermentation. Many pathways and enzymes associated with fermentation metabolisms in this alga are just being defined, and there is almost nothing known about the mechanisms by which these pathways are regulated and the trafficking of fermentation products among the different compartments in the cell. In general, photosynthetic algae appear to have a broad inventory of fermentative enzymes and, based on evidence discussed in this review, it appears that anaerobic respiration among eukaryotic algae is comparatively rare while anaerobic fermentation is widespread. Most enzymes for fermentative metabolism in the algae, often inferred from genomic and metabolic studies, have not been characterized biochemically. Expression patterns of genes encoding these enzymes and the biochemical properties of these enzymes and pathways need further characterization in a broader spectrum of algal systems. In addition, the diversity of end products that the various algae can synthesize during anaerobic fermentation is still mostly unknown. This information will be critical for developing a clear understanding of the metabolic diversity both within and among the different algal groups and the ways in which fermentation pathways have evolved and are shaped by environmental conditions. Finally, fermentation metabolism in the algae appears to represent a significant ecological component of carbon flux in soils (and sediments) that has a strong impact on its content of organic acids, alcohols, and H_2_; more focus on fermentation in the future is likely to unmask a relatively unexplored aspect of carbon cycling in the environment.

## Conflict of Interest Statement

The authors declare that the research was conducted in the absence of any commercial or financial relationships that could be construed as a potential conflict of interest.
